# Discrimination of the chemotherapy resistance status of human leukemia and glioblastoma cell lines by MALDI-TOF-MS profiling

**DOI:** 10.1038/s41598-023-32608-2

**Published:** 2023-04-05

**Authors:** Martín Ledesma, Daniela Poodts, Sofía Amoia, Silvia Hajos, Ariela Fundia, Carlos Vay, Matías Pibuel, Silvina Lompardía

**Affiliations:** 1 Unidad de Conocimiento Traslacional, Hospital de Alta Complejidad del Bicentenario Esteban Echeverría, San Martín 504, B1842 Monte Grande, Provincia de Buenos Aires Argentina; 2 Cátedra de Inmunología, Departamento de Microbiología, Inmunología, Biotecnología y Genética, Facultad de Farmacia y Bioquímica, Universidad de Buenos Aires (UBA), Junín 956, C1113 Buenos Aires, Argentina; 3 Instituto de Estudios de la Inmunidad Humoral (IDEHU), UBA-Consejo Nacional de Investigaciones Científicas y Técnicas (CONICET), Junín 956, C1113 Buenos Aires, Argentina; 4 Instituto de Medicina Experimental (IMEX)-CONICET, Academia Nacional de Medicina, José Andrés Pacheco de Melo 3081, C1425 Buenos Aires, Argentina; 5 Laboratorio de Bacteriología, Departamento de Bioquímica Clínica, Facultad de Farmacia y Bioquímica, Hospital de Clínicas “José de San Martín”, UBA, Av. Córdoba 2351, C1120 Buenos Aires, Argentina

**Keywords:** Proteomic analysis, Cancer

## Abstract

Chemotherapy mistreatment is partially due to a lack of rapid and reliable tools to discriminate between sensitive and resistant phenotypes. In many cases, the resistance mechanism is not fully understood, contributing to the diagnostic tools' absence. This work aims to determine the capacity of MALDI-TOF-MS profiling to discriminate between chemotherapy-resistant and sensitive phenotypes in leukemia and glioblastoma cells. A multivariate analysis of two therapy-resistant leukemia cell lines (Ki562 and Kv562) and two TMZ-resistant glioblastoma cell lines (U251-R and LN229-R) and their sensitive counterparts was performed. In this work, we first show MALDI-TOF-MS patterns analysis ability to differentiate these cancer cell lines by their chemotherapy-resistant status. We present a rapid and inexpensive tool that would guide and complement the therapeutic decision.

## Introduction

Survival and quality of life for cancer patients remain poor despite treatment improvements made over the last few years. It is well known that many chemotherapy drugs have adverse effects that impair the patient quality of life. Currently, patients receive chemotherapy based on the standard of care established, in many cases without a study of their particular tumor sensitivity. Consequently, sometimes patients are treated with ineffective drugs, which give poor benefits and diminish their quality of life. Therefore, developing strategies that guide therapeutic decisions in a personalized manner is required.

Up to our knowledge, there are no rapid and point-of-care diagnostic methods to assess chemotherapy resistance. Over the past years, the emergence of "omics" approaches has improved the study of multiple diseases, including cancer. Matrix-assisted light desorption ionization-time of flight mass spectrometry (MALDI-TOF-MS) is a robust tool for studying proteins in complex biological samples with high fidelity^1–3^. Notably, this technology is highly cost-effective in clinical microbiology for identifying bacteria^4^, and it is becoming increasingly available in the clinical diagnosis ground, even in resource-constrained countries.

Interestingly, only three groups analyzed mammalian cells through this procedure. In these works, the MALDI-TOF-MS tool was employed with different aims as the exclusion of cross-contamination in cell lines or the classification of cancer cells^5–7^. Taking this into account, we decided to use human chronic myeloid leukemia (CML) and glioblastoma (GBM) cell lines as models of malignancies in which the absence of personalized medicine tools directly impacts patient survival and quality of life.

CML is a myeloproliferative syndrome with an incidence of 1–2 cases over 100.000 people/year, representing about 15% of all adult leukemia cases. This pathology has different phases (chronic, accelerated, and blastic) with its own clinical characteristics. The main genomic feature is the Philadelphia chromosome, the product of a reciprocal translation between chromosomes 9 and 22 [t(9;22)(q34;q11)], which generates a fusion gene that encodes a constitutively activated kinase BCR-ABL^8^. This enzyme is responsible for leukemogenesis; thus, the first-line therapies are based on tyrosine kinase inhibitors (TKI) such as imatinib, nilotinib, dasatinib, bosutinib and ponatinib^9–11^. Although imatinib is the first-line treatment, in the face of therapeutic failure, the recommendation is to continue with other TKI. Therefore, the ability to anticipate the imatinib-resistant phenotypes would aid in the decision-making process regarding which drug to be implemented, which could positively impact the overall response to treatment. It is worth noting that, even though these drugs are highly effective, their long-term use favors the selection of resistant cells conducing to therapeutic failure^10–12^. The resistant mechanisms have been classified into BCR-ABL dependent and independent. The first includes active site mutations, gene amplification, and enzyme overexpression, while the second is mediated by the over-activation of PI3K and MAPK signaling pathways and efflux bombs activity^10,13–16^. The Kv562 cell line used in this work present BCR-ABL independent mechanisms as we previously demonstrated^17,18^.

Regarding GBM, this neoplasm is the most frequent and aggressive type of primary brain tumor^19^. The current therapy includes surgical resection, radiotherapy, and Temozolomide (TMZ) as first-line chemotherapy^20,21^. Notably, over 50% of patients are refractory to TMZ, mainly due to overexpression of O-6-methylguanine-DNA methyltransferase (MGMT) enzyme, although other players were found to be involved in this therapeutic resistance^22–24^. Moreover, the myeloid and hepatic toxicity caused by TMZ limit its use and negatively impact several patients quality of life^25,26^. Even though the FDA has approved three other agents (Lomustin, Carmustin, and Bevacizumab) for GBM treatment, patient survival remains at 14.6 months after diagnosis^21^.

In this work, we evaluate the protein profile of two therapy-resistant leukemia cell lines (Ki562 and Kv562) and two TMZ-resistant glioblastoma cell lines (U251-R and LN229-R) and their sensitive counterparts and perform a multivariate analysis. As a result, we aim to analyze if MALDI-TOF-MS analysis allows the differentiation between chemotherapy-sensitive and resistant cancer cell lines based on their protein profiles.

## Materials and methods

### Reagents

Drugs used were imatinib (Novartis, Switzerland) and temozolomide (TMZ, LKM, Argentina). RPMI 1640, DMEM, L-glutamine, streptomycin, penicillin, and XTT were purchased from Invitrogen (Waltham, Massachusetts, USA). Phenazine methosulfate (PMS), formic acid, acetonitrile, matrix solution (alpha- cyano-4-hydroxycinnamic acid matrix), and trifluoroacetic acid were purchased from Sigma-Aldrich (Saint Louis, Missouri, USA).

### Cell culture

**Figure 1:**
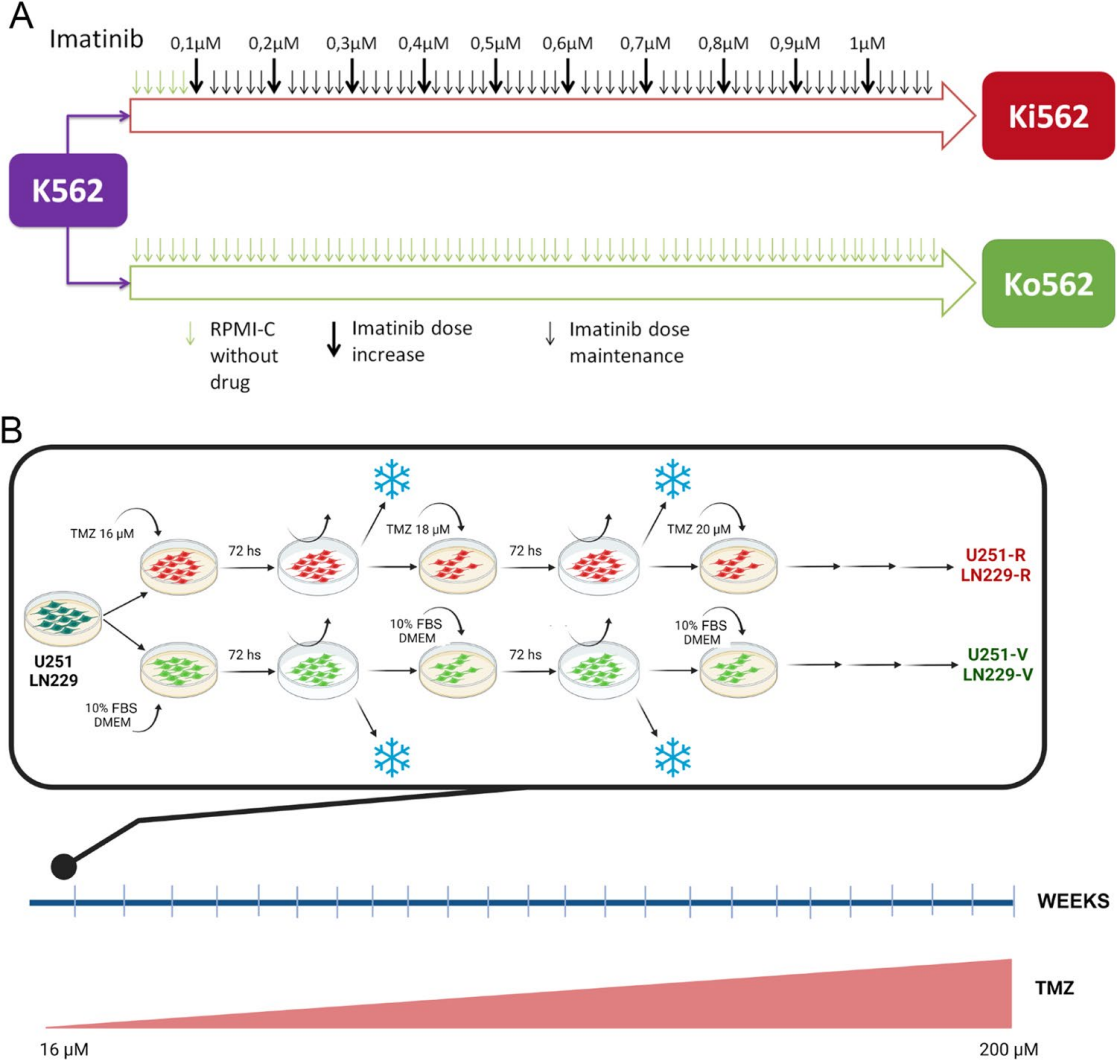
Establishment of resistant cell lines. (**A**) Ki562 cells were obtained by culturing K562 cells in the presence of increasing doses of imatinib. In parallel, K562 cells were grown without drug selection pressure, in order to accumulate the same level of culture aging. The latter was named Ko562. (**B**) U251 and LN229 were cultured with increasing doses of TMZ, obtaining U251-R and LN229-R cells, respectively. Both cell lines were also grown without TMZ pressure obtaining U251-V and LN229-V as aging controls.

Human CML cell lines K562, Ko562, Ki562, and Kv562 were grown in suspension cultures at 37 °C in a 5% CO2 atmosphere with RPMI 1640 supplemented with 10% heat-inactivated fetal bovine serum (FBS), 2 mM L-glutamine, 10 mM HEPES buffer, 5 × 10^−5^ M 2-mercaptoethanol, 100 µg/ml streptomycin and 100 IU/ml penicillin (RPMI-C). Kv562 cells were previously obtained by incubating K562 cells with increasing doses of vincristine and were grown in the presence of 150 ng/ml (162 nM) of this drug^17^. The resistance developed is mediated by both P-glycoprotein (Pgp) and PI3K over-activation^17,18^. It is worth noting that such mechanisms are also involved in imatinib resistance, being BCR-ABL independent mechanisms^16,27^. Ki562 cells were cultured in the presence of 1 µM Imatinib and were obtained as described in the results section (Fig. 1A), considering previous reports^28,29^.

The LN229 and U251 human GBM cell lines (gently provided by Dr. C. Perez-Castro and M. Candolffi, respectively) were grown in adherent cultures at 37 °C in a 5% CO2 atmosphere with DMEM supplemented with 10% heat-inactivated FBS, 2 mM L-glutamine, 100 µg/mL streptomycin and 100 IU/mL penicillin (DMEM-C), as we previously described^30^. Both resistant cell lines U251-R and LN229-R and both aged controls U251-V and LN229-V were obtained as described in the results section (Fig. 1B) considering previous reports^31,32^. In addition, U251-R and LN229-R cells were grown in the presence of 200 µM TMZ.

### Metabolic activity

An XTT assay was performed for metabolic activity as we previously described^30,33,34^. Briefly, 3 × 10^3^ GBM cells/well or 2.5 × 10^5^ CML cells/ml were seeded in 96-well plates and treated with TMZ for 72 h or Imatinib for 48 h. After treatment, 25 μl of an XTT solution (1 mg/ml) containing PMS (7.5 µg/ml) was added to the culture medium (100 μl), and cells were incubated for two additional hours at 37 °C in a 5% CO2 atmosphere. After incubation, the absorbance (Ab) was read at 450 and 620 nm using a microplate reader (Multiscan Ex, Absorbance Microplate Reader, Thermo Electron Corporation, China).

The corrected absorbance was calculated as Ab (treated)450 – Ab (treated)620, as we previously described^30,33,34^.

Cell lines were processed by biological and experimental triplicate. The metabolic index was calculated for each drug concentration as a percentage of the treated averaged absorbance relative to the basal averaged absorbance. Half-maximal effective concentration (EC50) was used because not all entities ultimately reached 50% of inhibition in the range of drug concentration used. To determine EC50s using our data, we perform a non-linear (log-logistic function) regression using the drc R package, specifically for analyzing dose–response curves^35^. The model was used to estimate four parameters: the maximum value, the minimum value; the EC50; and the Hill coefficient. Finally, we used these estimated parameters to calculate the fitted viability values for each concentration value.

It is worth noting that this assay was not carried out in Kv562 cells since they were previously established and their resistance to imatinib has already been demonstrated in our previous reports^18,36,37^.

### Acquisition of MALDI-TOF-MS spectra

A fast MALDI-TOF-MS approach was applied to mammalian cell lines based on whole-cell extract analysis. 1 × 10^6^ cells were seeded in a 100 mm plate and incubated for 24 h for the correct attachment of cells. Then, the medium culture was changed, and cells were incubated for an additional 24 h, harvested, centrifuged, and resuspended in 500 ml of PBS. This procedure was carried out in triplicate (Biological replicate), and MALDI-TOF-MS analysis was performed for each. Aliquots were centrifuged (10.000 Relative Centrifugal Force (RCF), 5 min), then the supernatant was discarded, and cell pellets were homogenized with 25 μl of 100% formic acid (Sigma Aldrich) and 25 μl of 100% acetonitrile (Sigma Aldrich). After centrifugation (10.000 RCF, 5 min), 1 μl of the supernatant was loaded by quadruplicate (Experimental replicate) onto each spot of the MALDI metal plate and overlaid with 1 µl of matrix solution (alpha- cyano-4-hydroxycinnamic acid matrix in 50% acetonitrile and 2.5% trifluoroacetic acid (Sigma-Aldrich, St. Louis, MO, USA)). Duplicates or triplicates were acquired for each spot (Technical replicate). Continuous mass spectra (Ms) were obtained with a Microflex LT mass spectrometer (Bruker Daltonics, Inc., Billerica, MA, USA) (ionization mode: LD, laser shots: 20, laser repetition: 60) within a mass range of 2000–20,000 Da. Ms were internally calibrated and controlled daily using Escherichia coli ribosomal proteins (Bacterial Test Standard, Bruker Daltonik Gmb).

### MALDI-TOF-MS data pre-processing

Ms were read as fid/aqus files with MALDIquantForeign (v0.10)^38^ and processed using MALDIquant (v1.16.2) R package^39^. Spectra were square root-transformed and smoothed, employing the Savitzky-Golay algorithm. Then, they were baseline-corrected, applying the Statistics-sensitive Non-linear Iterative Peak-clipping algorithm (SNIP) process across 50 iterations^40^. The peak detection was carried out by a function that estimates the noise of mass spectrometry data by computing the median absolute deviation (MAD). The signal-to-noise ratio (SNR) was set up in 3, with a half window size of 40 and a tolerance of 0.2. Peaks that occur in less than 50% of spectra were discarded. Finally, spectra were averaged by their technical replicates. MALDI-TOF-MS data were categorized with the Binda R package^41^. Programmed peak selection was performed in the entire dataset seeking biomarkers of chemotherapy susceptibility status by the Binary Discriminant Analysis (BDA) algorithm^41^. The best-extracted peaks were then used to run a hierarchical k-means clustering-Principal Component Analysis (PCA) with the Factoextra R package^42^. The binary distance was used to measure dissimilarity between observations, ward.D2 was the agglomeration method used, and k was set to 2 or 3, considering the displayed levels in comparison^43^.

## Results

1. Establishment of the resistant cell lines

First, it was decided to select different cell lines which present resistance to the first-line drugs. With this purpose, K562 cells were grown in the presence of increasing doses of imatinib from 0.1 µM up to 1 µM (for 4 months), obtaining Ki562 cells according to previous reports^28,29^. Control cells derived from K562, Ko562, were cultured in parallel but without the selection pressure of imatinib (Fig. 1A). It is important to highlight that Kv562 cells were previously established in a similar way to Ki562 cells but using vincristine as selection pressure. This cell line presents multidrug resistance due to Pgp activity (an efflux pump), as well as PI3K overactivation, and both are BCR-ABL independent resistance mechanisms^17,18,36^.

In the same way, U251 and LN229 cells were grown with TMZ from 16 µM up to 200 µM (for 6 months), obtaining U251-R and LN229-R cells (Fig. 1B) in concordance with previous reports^31,32^. Control cells derived from both cell lines, U251-V and LN229-V, were cultured in parallel but without the selection pressure of TMZ (Fig. 1B).

2. Effect of imatinib and TMZ on metabolic activity in the different cell lines

To confirm the acquisition of the resistant phenotype, the metabolic activity after imatinib or TMZ treatment was evaluated by XTT assay.

**Figure 2:**
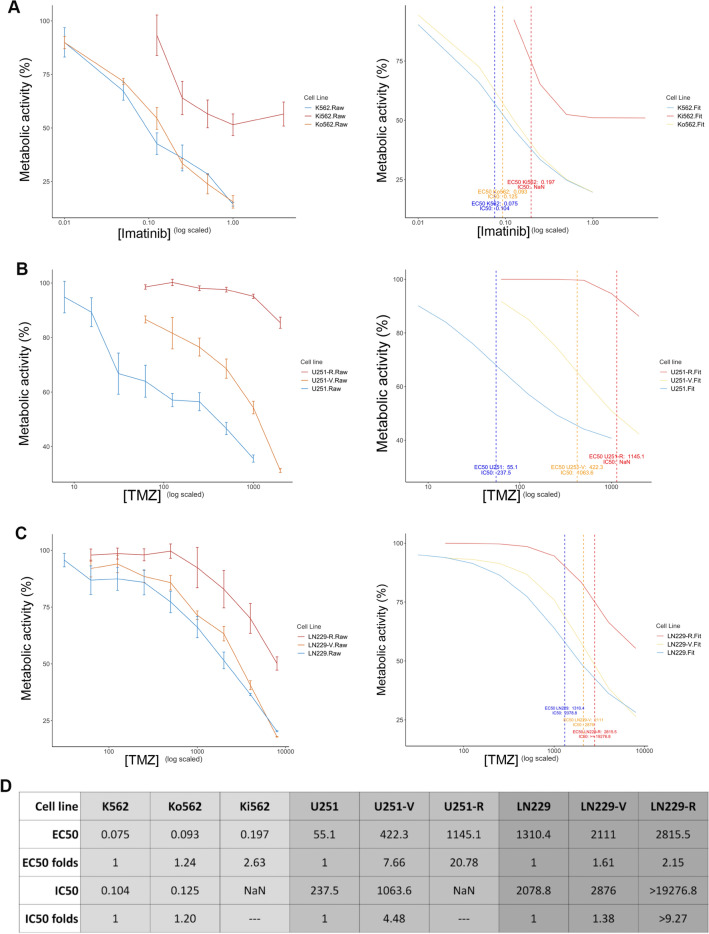
Effect of first-line drugs on metabolic activity. The different cell lines were treated with (**A**) RPMI-C or Imatinib (K562, Ko562, and Ki562), (**B**) DMEM-C or TMZ (U251, U251-V, and U251-R) or (**C**) DMEM-C or TMZ (LN229, LN229-V, and LN229-R) for 48 h (CML cells) or 72 h (GBM cells) and metabolic activity were evaluated by XTT assay. Left panel shows Raw data while right panel shows Fit data. (**D**) Comparison of the IC50 and EC50 obtained to each model. NaN stands for not a number, and the meaning is that the curve did not reach the 50% inhibition needed to compute de IC50.

Figure 2A and D show that the EC50 of imatinib in Ki562 cells is 2.11 folds and 2.62 folds higher than Ko562 and K562, respectively. Moreover, IC50 could not be calculated in Ki562 cells, while it was 0.125 and 0.104 µM in Ko562 cells and K562 cells, respectively. It is worth highlighting that the resistance of the Kv562 cell line to the effect of imatinib was previously demonstrated^18,36,37^. In these works, we evaluated cell proliferation (by ^3^H-Thymidine uptake), demonstrating that the IC50 of imatinib in Kv562 cells is 5.11 folds higher than K562

.

Figure 2B and D show that U251-R cells present an EC50 20.7-folds higher than U251 cells. Interestingly, due to an increase in the number of passages of culture, the U251-V cell line increased its resistance to TMZ by 7.6 times concerning the U251 cells. Similar results were obtained for LN229 cells, as shown in Fig. 2C and D. LN229-R cells present an EC50 2.15 folds higher than LN229, while in LN229-V it is 1.61 folds.

These results confirm that we successfully obtained a new imatinib-resistant CML cell line and two TMZ-resistant GBM models. In addition, the aging of the cultures, even without the selection pressure of TMZ, seems to induce a low resistance to the first-line drug in the in vitro GBM models analyzed.

3. MALDI-TOF-MS analysis

A statistical analysis pipeline was implemented to test if MALDI-TOF-MS data could discriminate the chemotherapy susceptibility status of different cancer cell lines. First, a supervised classification by the Binary Discriminant Analysis (BDA) was implemented, searching for peaks that best differentiated the groups. A subset of the best-ranked peaks was used for conducting a hierarchical k-means clustering-PCA. The results for each model are described below.

A **CML model**

Four leukemia cell lines were considered for MALDI-TOF-MS analysis: K562, Ko562, Kv562, and Ki562. As previously described, K562 is the Imatinib-sensitive cell line, Ko562 is the aged control cell line (which is also sensitive to imatinib although its EC50 increased by 24% regarding to K562), while Ki562 and Kv562 are two different chemotherapy-resistant cells to Imatinib. Table 1 contains detailed information about the experimental and technical replicates obtained throughout the experiments. In summary, a total of 77 Ms were obtained for leukemia cell lines, and the composition is the following: 16 (K562) + 22 (K562) + 16 (Ki562) + 23 (Kv562). After the averaging at the technical replicate, 40 averaged Ms were obtained: 9 (K562) + 11 (K562) + 8 (Ki562) + 12 (Kv562). These averaged Ms were further analyzed.

**Table 1:** Detailed information about experimental and technical replicates obtained with all the cell lines under investigation.

Cell line	Experimental replicates (Obtained/Total)	Technical replicates (Obtained/Total)
K562	9/12	16/18
Ko562	11/12	22/22
Ki562	8/12	16/16
Kv562	12/12	23/24
U251	12/12	36/36
U251-V	11/12	32/33
U251-R	12/12	36/36
LN229	14/15	40/42
LN229-V	14/15	41/42
LN229-R	15/15	44/45

i) Comparison between sensitive K562, Ko562, and resistant CML cells

**Figure 3:**
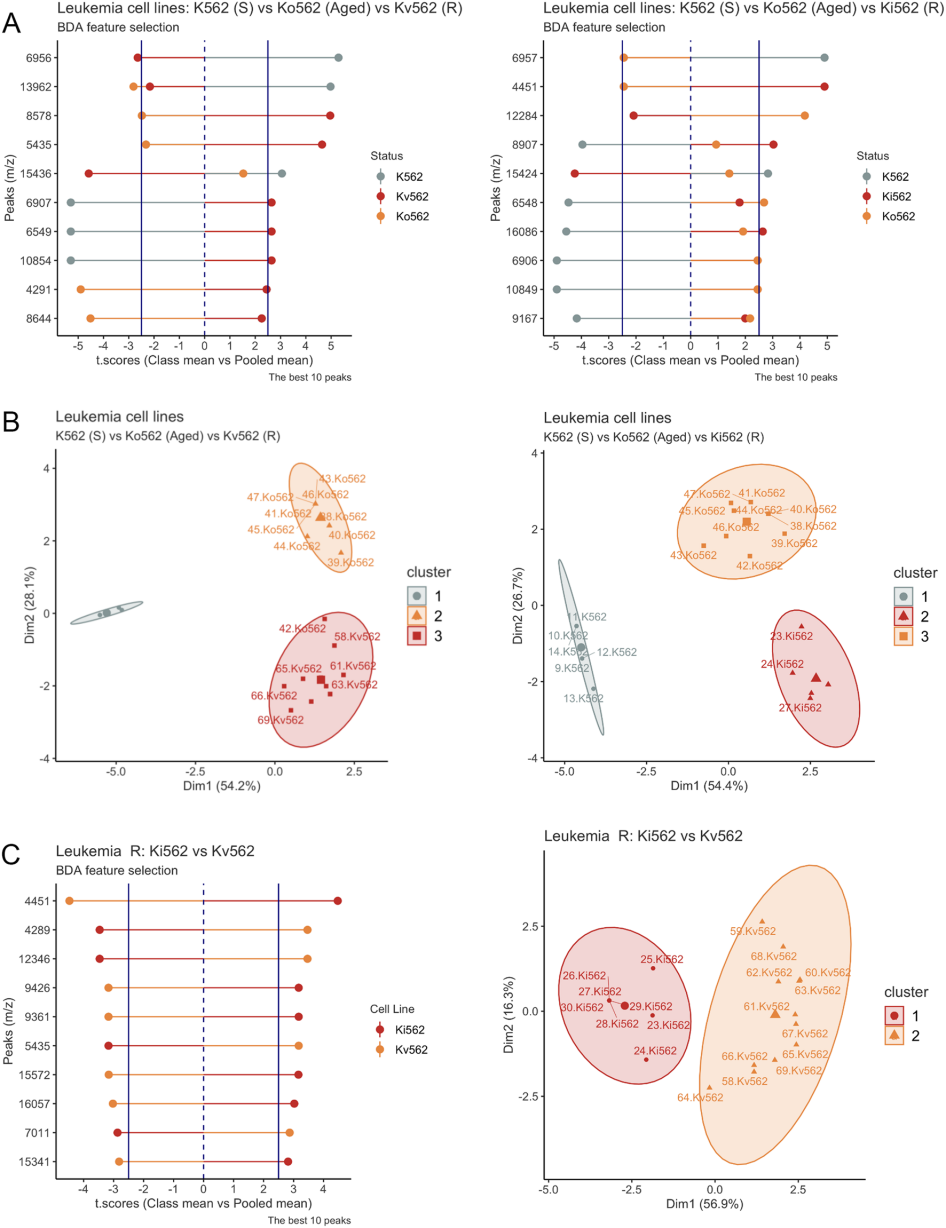
MALDI-TOF-MS analysis for the CML model. (**A**) Comparison between K562 (sensitive)/Ko562 (Aged-sensitive)/Kv562 (resistant) (left panel) and K562/Ko562/Ki562 (resistant) (right panel) for the selection of discriminant peaks by the BDA algorithm. The algorithm outputs the t.score (group means vs. pooled mean), which is graphed in the x-axes of both graphs, and the peaks (m/z) are plotted on the y-axis. The sign of the t.score indicates the presence (positive) or the absence (negative) of that peak in that group. The groups are indicated with different colors. The differences have a 95% significance when the value of the t.score is equal to or greater than 2.5 (increased) and equal to or less than − 2.5 (diminished). (**B**) Spectra pattern comparison between K562/Ko562/Kv562 (left panel) and K562/Ko562/Ki562 (right panel), was performed by the Hierarchical k-means clustering with a Principal Component Analysis visualization (HKmC-PCA). Each point on the graph corresponds to each experimental replicate. The label indicates an ID number of the spectrum and the cell line name. The spectra patterns were grouped in 3 clusters represented by different geometric symbols and colors. Lastly, the ellipses of each cluster represent 95% of confidence from the centroid, considering a normal distribution multivariate. (**C**) Comparison between the resistant cells Kv562/Ki562. Left panel: discriminant peaks by the BDA algorithm and right panel: HKmC-PCA.

Cell lines Ms patterns of K562-Ko562-Kv562 and K562-Ko562-Ki562 were compared separately. Additionally, both resistant phenotypes were analyzed against the K562 and Ko562 cell lines. Figure 3A shows the result of the supervised BDA algorithm searching for the peaks that best differentiated between these three groups. Figure 3B shows the result of the unsupervised statistical model, the hierarchical k-means clustering Principal Component Analysis (Hkmc-PCA), performed to test the capacity of the prior selected peaks to separate the Ms in separated and homogeneous clusters.

In both cases, clusters appeared as three non-superimposed groups, and the first two principal components explained more than 70% of the variation, which means a significant cluster separation (Fig. 3B). In the K562-Ko562-Ki562 comparison, cluster homogeneity reached 100% for all three groups. Consequently, Ms data allows a perfect separation of these cell lines. In the case of the K562-Ko562-Kv562 comparison, cluster 1 (K562) y 2 (Ko562) attained 100% homogeneity, while cluster 3 (Kv562) had 92% of homogeneity. In this last case, a Ko562 experimental replicate falls within cluster 3. Nevertheless, considering the basal K562 vs. the resistant Kv562, separation is still flawless.

ii) Comparison between resistant CML cell lines

Then we decided to analyze and compare Kv562 and Ki562 resistant cell lines Ms patterns. Clusters appeared as two non-superimposed groups, and the first two principal components described more than 70% of the deviation, representing a robust cluster split (Fig. 3C). Cluster homogeneity was 100% for both groups. Accordingly, Ms data allows a perfect separation of these cell lines.

B **Glioblastoma model**

i) Comparison between sensitive, low,and high-resistant GBM cells

U251 (TMZ sensitive), U251-R (high resistance to TMZ), and U251-V (low resistance to TMZ) were analyzed by biological triplicate, experimental quadruplicate, and technical triplicate (Table1). In summary, a total of 104 Ms were obtained for GBM cell lines, and the composition was: 36 (U251) + 32 (U251-V) + 36 (U251-R). After the averaging at the technical replicate, 35 averaged Ms were obtained: 12 (U251) + 11 (U251-V) + 12 (U251-R). These averaged Ms were further analyzed.

**Figure 4:**
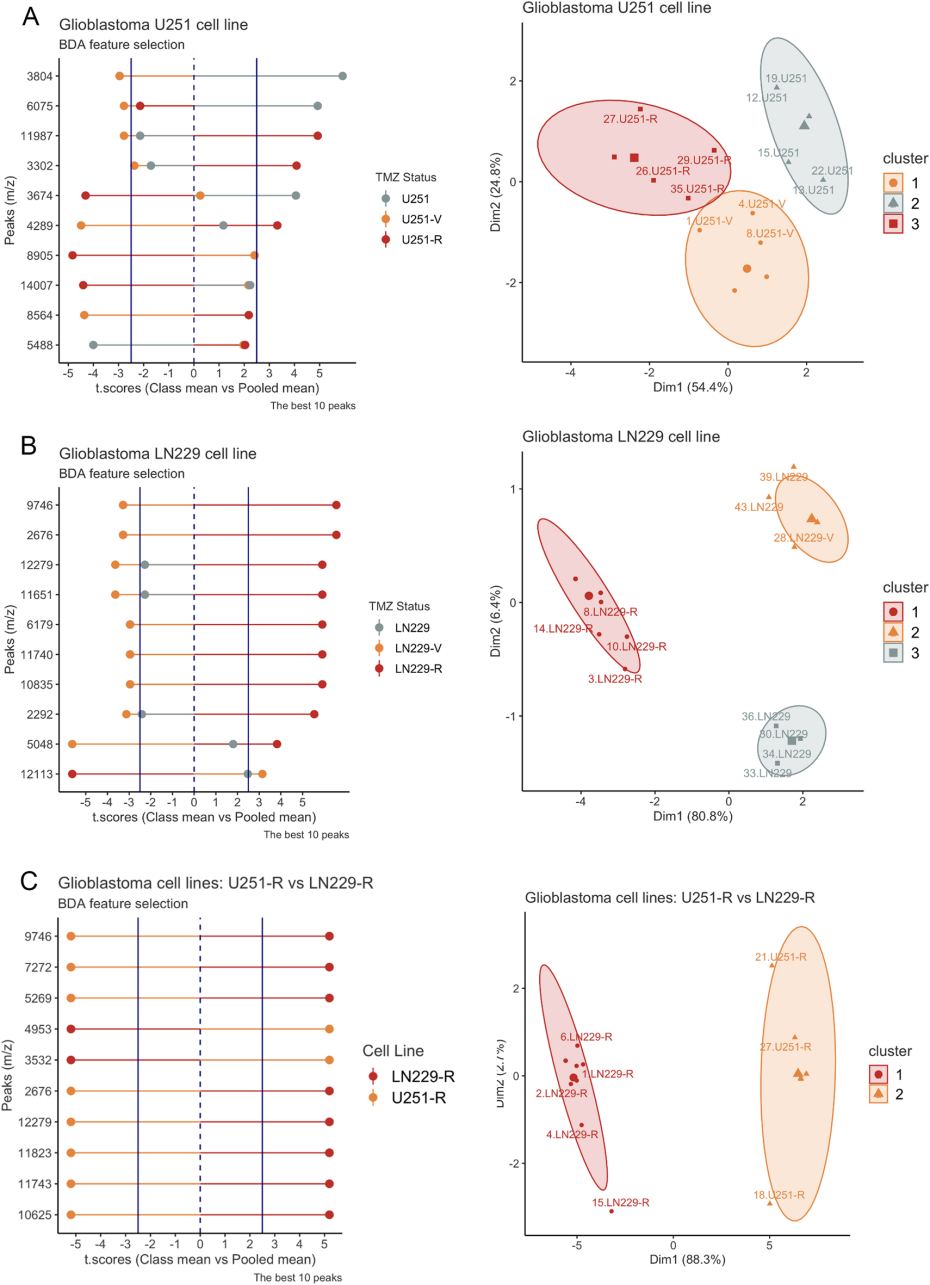
MALDI-TOF-MS analysis for the GBM model. Left panel: comparison between (**A**) U251/ U251-V/U251-R, (**B**) LN229/ LN229-V/LN229-R, and (**C**) U251-R/LN229-R for the selection of discriminant peaks by the BDA algorithm. The algorithm outputs the t.score (group means vs. pooled mean), which is graphed in the x-axes of both graphs, and the peaks (m/z) are plotted on the y-axis. The sign of the t.score indicates the presence (positive) or the absence (negative) of that peak in that group. The groups are indicated with different colors. The differences have a 95% significance when the value of the t.score is equal to or greater than 2.5 (increased) and equal to or less than − 2.5 (diminished). Right panel: Spectra pattern comparison between (**A**) U251/ U251-V/U251-R, (**B**) LN229/ LN229-V/LN229-R, and (**C**) U251-R/LN229-R, was performed by the Hierarchical k-means clustering with a Principal Component Analysis visualization (HKmC-PCA). Each point on the graph corresponds to each experimental replicate. The label indicates an ID number of the spectrum and the cell line name. The spectra patterns were grouped in 3 clusters represented by different geometric symbols and colors. Lastly, the ellipses of each cluster represent 95% of confidence from the centroid, considering a normal distribution multivariate.

Clusters appear as three non-superimposed groups, and the first two principal components described more than 70% of the variation, which means an effective cluster separation (Fig. 4A). Cluster homogeneity reached 100% for all three groups. Thus, Ms data allows a perfect separation of these cell lines.

LN229 (TMZ sensitive), LN229-R (high resistance to TMZ), and LN229-V (low resistance to TMZ) were analyzed by biological triplicate, experimental quintuplicate, and technical triplicate (Table 1). In summary, a sum of 125 Ms was obtained for LN229 GBM cell lines, and the composition was: 40 (LN229) + 41 (LN229-V) + 44 (LN229-R). After the averaging at the technical replicate, 43 averaged Ms were obtained: 14 (LN229) + 14 (LN229-V) + 15 (LN229-R). These averaged Ms were further studied.

Clusters appear as three non-superimposed groups, and the first two principal components described more than 70% of the variation, which means an effective cluster separation (Fig. 4B). In this case, clusters 1 (LN229) and 2 (LN229-R) attained 100% homogeneity, while cluster 3 (LN229-V) had 82% of homogeneity (14 LN229-V replicates and 3 LN229). LN229-R replicates appear as a separate group, emphasizing the approach's usefulness.

ii) Comparison between resistant GBM cell lines

Then we also analyze and compare U251-R and LN229-R cell lines' Ms patterns. Clusters appeared as two non-superimposed groups, and the first two principal components described more than 70% of the deviation, representing a robust cluster separation (Fig. 4C). Cluster homogeneity was 100% for both groups. As expected, Ms data allows a perfect separation of these cell lines.

## Discussion

The development of tools that facilitate and promote the personalization of therapy in oncology is a necessity. In this work, we demonstrated that the MALDI-TOF-MS pattern analysis, in a standard experimental setting for microbiological bio-typing, helped discriminate between chemotherapy-sensitive and resistant cell lines in both CML and glioblastoma models. Other works have reported using MALDI-TOF-MS to classify human tumor cells or to evaluate the cross-contamination of different cell lines^5–7^. However, this work demonstrates for the first time the ability of this tool to subtype CML and GBM cells according to their degree of first-line drug response. The ensemble between supervised (BDA) and unsupervised (Hkmc-PCA) algorithms result in prominent profiling of the chemotherapy susceptibility status. The first algorithm ranks the peaks in order of their group discrimination importance, and the second one visualizes the power of the former best-selected peaks to discriminate observations in separate and homogeneous clusters.

Our results show that chemotherapy-resistant and sensitive cell lines present a differentiable MALDI-TOF-MS pattern, making this technology a potential tool for evaluating chemotherapy susceptibility. Currently, two of the critical issues in cancer are the failure in the diagnosis and treatment. Therefore, these results would be the basis for translating this technology towards improving the prognosis and quality of life of patients with tumor pathologies. In this way, knowing each patient's susceptibility to chemotherapy would make it possible to administer the best chemotherapy option, with better results and fewer unnecessary adverse effects.

Furthermore, the methodology could also differentiate two distinctive leukemia-resistant phenotypes (Ki562 and Kv562) and two GBM-resistant phenotypes suggesting the potential use of this technology as personalized medicine tool. As we previously introduced, the mechanisms of resistance to imatinib can be dependent or independent of BCR-ABL. Kv562 cells present high Pgp activity and overactivation of PI3K, while Ki562 cells present an overexpression of BCR-ABL (recently determined by western blot, data not shown). Therefore, each resistant cell line represents the different types of resistance to imatinib. This observation gives relevance to the in vitro model used.

Interestingly, aged culture cell lines (Ko562, U251-V, and LN229-V) exhibit characteristic Ms patterns, different than their sensitive and resistant counterparts, showing EC50s higher than the sensitive cells in both tumor models. This effect is more evident in the GBM model. This biological advantage could be due to the long-term culture and the elevated mutational rate characteristic of tumor cells. This observation could be in accordance with the bad prognosis of the patients after late diagnoses. In this sense, the longer growth time would give an opportunity for the accumulation of mutations and selection of cells with better fitness, favoring the malignancy.

This high throughput technology is currently available in several healthcare facilities for microbiological biotyping^4^. Therefore, its aid in the decision-making process in cancer treatment would pose an unprecedented repositioning of this technology. Its good availability and quickness could directly impact therapeutic success rate and thus improve patients' health and quality of life. In this way, in the case of GBM, obtaining a resistant cell pattern could re-direct the treatment to radiotherapy or try the few therapeutic alternatives available, avoiding the TMZ negative health cost. In the case of CML, an imatinib-resistant pattern could confer a signal to employ another TKI drug improving the therapeutic response of the patient, as well as preventing selective pressure enhancement.

All in all, this work paves the way for future studies considering this technology's use in the oncology therapeutic decision. Although the tests were carried out using cell line models, the results were consistent, and we hope to continue the study in patient samples in order to translate this tool to the clinic. The ensemble between supervised (BDA) and unsupervised (Hkmc-PCA) algorithms results in a prominent profile of susceptibility status to first-line chemotherapy. In addition, the equipment used in this work is the same that is already used for bacterial biotyping, facilitating its potential translation since many clinical laboratories already have the equipment. Another advantage is the brief processing time which is in the hour scale. In this way, our work opens the possibility of improving therapy using this technique, promoting personalized medicine, and impacting the quality of life of cancer patients.

## Conclusion

MALDI-TOF-MS spectra patterns analyzed by multivariate strategies have proved to be a valuable tool for discriminating leukemia and glioblastoma cell lines regarding their chemotherapy susceptibility status. This work is an ideal starting point for further research and validation of a possible point-of-care MALDI-TOF-MS protocol for tumor profiling.

## Data Availability

The datasets used and/or analyzed during the current study are available from the corresponding author on reasonable request.
